# The New-England Journal of Medicine and Surgery, and the Collateral Branches of Science

**Published:** 1813-01

**Authors:** 


### CRITICAL ANALYSIS
### *OF RECENT PUBLICATIONS*
### IN THE
### DIFFERENT BRANCHES OF PHYSIC, SURGERY, AND MEDICAL PHILOSOPHY.

*The New-England Journal of Medicine and Surgery, and the collateral Branches of Science.* Conducted by a number of Physicians. No. II. Vol. I. Published quarterly. Boston. April, 1812.

IT affords us sincere pleasure to notice the appearance of this transatlantic work. The first Number has not reached us; and, for that which we now proceed to notice, we are indebted to a much-valued friend. The design of this Journal is similar to that of our own; the first part being devoted to original papers; the second to a review of new publications; and the remainder to medical intelligence. In this place, our business is with the first part of the Journal. It begins with the continuation of a paper by Dr. Jackson,‡ on

‡ It is not doing the author justice, to notice only part of his communication; but, as it contains some interesting matter, and as few of our readers can obtain the American Journal, we trust we shall be excused for extracting as much from it as our limits will admit.

the the morbid effects of dentition, more particularly with reference to the diseases of teething children in summer and autumn. The author commences this part of his dissertation with an enumeration of the causes of *Cholera Infantum*. These he chiefly refers to dentition, the season, improper food, restraint from exercise in the open air, and an impure atmosphere. On each of these he briefly comments. Dentition acts as a remote cause by the irritation which it excites; but, as the first teeth, viz. the two middle incisors of the lower jaw, which protrude through the gums, are less frequently attended with pain or irritation, the disease, according to the author's observation, seldom occurs before the eighth or ninth month. Likewise it rarely commences in children who are past eighteen months.

"At this age infants have still a number of the temporary or milk-teeth, remaining within the gums; on an average they have six or eight teeth in this situation, and the passage of these teeth is often attended with some disorder of the primæ viæ, and often with violent constitutional irritation. There seems to be two reasons why the cholera infantum does not more frequently affect children past eighteen months. One is, that children at this age are able to run about freely; and then they acquire vigor by exercise, while they are not easily confined in an impure atmosphere. The other is, that, at this age, in subjects previously healthy, the stomach becomes more capable of digesting food of various kinds."

"*The season.*—This disease occurs sometimes, though rarely, in April, May, and June; that is, in the spring season of our climate. But its frequent occurrence is in July, August, September, and October; that is, in summer and autumn. In winter the disease seldom commences; and, even in cases where it had existed with great severity, its violence abates in November."

These facts support the author's conclusion, that there is some cause operating in the summer and autumnal seasons, favorable to the production of the disease. What that agent is, or in what mode it acts, he does not attempt to explain. At those times also, serious acute diseases are more frequent than in other seasons of the year; especially idiopathic fevers, and affections of the chylopoietic viscera. These complaints, as well as cholera, the author remarks, have been more prevalent in his district, when the summer has been warm and dry, than when cold and moist; though he candidly admits, that his experience has not been of sufficient duration to authorise him to rely upon it with full confidence. The remaining remote causes are very briefly noticed, and afford no room for comments.

The author searches for the proximate cause of cholera infantum, in the history of the disease; the symptoms during life, and the appearances after death. It differs from

 diarrhœa, diarrhœa, described in the first Number of the Journal, by the following symptoms:

" Pain or uneasiness after taking any thing into the stomach, the frequent rejection of the food shortly after it is swallowed, increased thirst, irregularity in the actions of the bowels, and the retention in them of fœcal matter. But, above all, we notice in the cholera a prostration of strength, an emaciation, and a shrinking of the whole body, accompanied by febrile paroxysms.

" It appears then, that, although in the cholera, as in the diarrhœa, there exist dyspepsia and irritation of the chylopoietic viscera, that there probably exists something more; that there is discovered an irritability of stomach, and a severe constitutional affection, greater than are the ordinary attendants on dyspepsia, especially when not yet of long continuance. The dyspepsia, &c. in the diarrhœa, imply only debility of the stomach in the performance of its digestive functions; but, in the cholera, there appear effects greater than, and different from, those which commonly belong to dyspepsia from debility. What is the cause of those greater and peculiar effects, is learned in the examination of the body after death. In this examination it appears, that the mucous membrane of the stomach, and of that portion of the small intestines most immediately connected with the stomach, has been affected with inflammation.

" It is then to this inflammation of the mucous membrane of the stomach and small intestines, that the peculiar phenomena of cholera infantum may be traced, in the same manner as those of dysentery have been found to be owing to a similar affection of the large intestines. It is when this inflammation supervenes in the autumnal diarrhœa of infants, that the disease assumes its serious and threatening aspect; and it is at this time that the popular remark is made, that now 'the canker has seized the bowels.' This inflammation is no doubt much more extensive and more severe during life, than it is found to be after a slow and lingering death. In different cases it varies in extent and violence, whence it happens that the symptoms appear more or less fully, and that there are cases intermediate between this disease and the diarrhœa, as before stated."

This subject will be resumed in a future Number.

The second paper in the collection contains three cases of organic diseases of the heart and lungs, by Dr. John Collins Warren. These we do not hesitate to insert entire, together with the learned author's observations upon them, because they form valuable additions to medical literature; and, as before stated, the American Journal, for the present, will pass through very few hands in this country.

" CASE I.—*Aneurism of the origin of the Aorta.*—Mr. ———, a gentleman of small stature, but uncommon muscular power, was affected in the autumn of 1808, with pains in the right shoulder, arm, and leg. His complaint being supposed to be rheumatism, he was bled and blistered. The pains often returned, and became so severe in the shoulder, in the spring of 1809, as to induce Dr. Eustis, a friend

 of of his family, and Dr. Bates, his physician, to make an examination of this part. When the breast was uncovered, Dr. Eustis observed with astonishment a small pulsating tumor on the right side of the thorax, between the second and third rib, at the distance of one or two inches from the sternum, Dr. Eustis pronounced to the friends of the patient, that his disease was of an incurable and fatal nature.

" Soon after, I examined this gentleman with Dr. Warren, senior, and Dr. Bates. We found the tumor very slightly projecting from the surrounding surface, possessing a strong pulsation, and a little tenderness on pressure. It was about two inches in diameter. The internal jugular vein of that side had a considerable pulsation. The pulse in the right arm was not sensibly different from that of the left, and neither of them changed from the healthy state; the patient informed us, that he was much troubled with dizziness and head-ach; that he had been formerly subject to enlargement of the hemorrhoidal veins with discharges of blood, which had not lately occurred, and that in other respects his health was unimpaired. We learnt from his friends, that he had accustomed himself to very severe and dangerous equestrian exercises, in which he took pride, and was very expert; and that he had long discovered signs of weakness in the thorax, especially in the year 1788, when he suffered greatly from the pressure of a bayonet-belt on that part, during his exercises in a military company.

" Although, at the period of our examination, he scarcely admitted the existence of symptoms which might indicate disease in an important organ, it was not long before the sufferings, connected with organic diseases in the heart, began to advance with slow, but formidable, steps. After his complaints had made some progress, they were suddenly arrested on the application of a large blister, and allowed him an interval of ease of four or five months' duration. In the month of March, 1810, after exposure to cold and moisture, his symptoms were increased in a very sudden and alarming manner. His respiration became laborious and suffocating, his cough incessant, and pain in the breast more violent than at any former time. He started often from sleep with a dread of suffocation, and was compelled to sit upright in bed. One of the most distressing symptoms was a difficulty in swallowing, which greatly increased in this paroxysm of disease. His cough was violent, and attended with a copious expectoration of whitish mucus. This paroxysm was alleviated; but he never had an easy day, nor a quiet night, afterward. The disorder occurred in fits of two or three days' duration. In the intermissions, he was comparatively comfortable, and able to attend to business. The symptoms scarcely changed afterward, except in degree, and in the increased frequency of their recurrence. The respiration became at last very laborious, and was attended with a loud noise. The cough more violent, expectoration greater, and tinged with blood. The right jugular vein was dilated enormously; while the carotid artery of that side apparently lost its pulsation. The pulse was weaker on the right than on the left side, and in the last paroxysm intermitted. This paroxysm occurred in August, 1811.

It continued about four days, and terminated with the appearances of suffocation.

"In the course of the disease, Dr. Bates frequently relieved the symptoms by blistering, by calomel with opium, and other medicines which promote expectoration. Dr. Warren employed bleeding and various narcotic substances, particularly stramonium and cicuta, with temporary advantage.

"*Dissection.*—On the day following the patient's death, I examined the body in presence of a number of medical gentlemen.

"The countenance was slightly bloated and livid. The extremities were not œdematous. We observed that the third rib on the right side was pushed out, at least the space of an inch. The projection was greatest at about two inches distance from the sternum. The skin over it was livid, and appeared thin, as if ready to burst. When the cartilages of the ribs had been divided, and two or three ribs sawed, we found it difficult to raise the sternum, which was discovered to adhere to a substance in the thorax. The ribs were very carefully separated, but not without tearing open this substance, and exposing a cavity. This we discovered to be a great tumor from the right side and posterior part of the aorta, at the root of the arteria innominata. This tumor had a narrow base, so as to leave half the circumference of the aorta uninjured. It pressed forward on the second and third ribs, and the right edge of the sternum, which had become carious. There was a separate tumor on the back part of the arch of the aorta, extending from the arteria innominata to the left carotid artery, of smaller size than the other. The latter involved the origin of the arteria innominata, which was placed on its superior and posterior portion. It extended to the spine, pressed on the trachea, and adhered to it at the bifurcation, and pressed also on the œsophagus. The upper and central part of the thorax was occupied by these two aneurisms, from the sternum to the spine. The cavities of both were filled with coagulated blood; yet not in such manner as evidently to interrupt the canal of the aorta, or of the great arteries of its curvature.* The heart was of a small size. The texture of the lungs was healthy, and not much filled with blood. The air-vessels of the lungs were crowded with a very white-colored mucus. In the right cavity of the thorax we saw about ten ounces of water, and five or six ounces in the cavity of the pericardium. The abdominal organs were sound, and their cavity without water.

"Case II.—*Opening in the mitral valve.*—A healthy female, 21 years of age, who had been married 5 years, and had two children, the last of which had been weaned a week, when she was taken ill: was suddenly attacked with an acute pain in the left foot, that continued a whole night, and subsided in the morning. On the afternoon of the following day, she was affected with an acute pain in the left shoulder, darting thence through the clavicle to the heart. Her skin was hot, face flushed, and pulse hard. Dr. Bean, who was

* A neatly-executed plate represents about half the extent of the largest aneurismal tumor.

called called to her, bled and blistered her without effect, but she was eventually relieved by the use of opium. The pain recurred at intervals afterward. About a week from the time of the first attack, she had a very severe chill, accompanied with extreme lividity of the face. These symptoms subsided in about half an hour, but recurred two or three times a-day afterward, and frequently terminated in fainting. A numbness of the left side, which she had experienced at first in a slight degree, increased very much. Her respiration became extremely difficult, and required her chest to be raised high in bed. Her sleep was interrupted by frightful dreams, during which she started up and screamed that she was suffocating. The pulse at this time was very irregular and intermittent. The heart palpitated violently. About six days before death, the legs swelled, and the pain in the shoulder subsided. She expired on the twentieth day from the first attack, with symptoms of suffocation. The fatal paroxysm invaded her in the manner described above. Her respiration was laborious, pulse scarcely perceptible, lips livid, and eyes wild and staring.

" *Dissection.*—The body was examined by Drs. Jackson and Bean. When the heart was opened, the mitral valve nearest the aorta was discovered to have an opening, through which one's finger might be passed. The edge of the opening was surrounded with a thick substance, which gave it the appearance of a fringe. The cavity of the thorax contained a large quantity of water. The appearance of the subject was unusually white, and generally œdematous.

" Case III.—*Disease of the Lungs, the symptoms of which much resembled those of organic diseases of the Heart.*—Sarah Collier, aged 27, was attacked, on the 28th of January, 1810, with profuse hœmorrhage from the lungs, and raised by coughing large quantities of florid blood. This attack was accompanied with severe pain in the left side of the chest, greatly increased upon forcibly inspiring. Her breathing was quick and laborious, and her pulse hard and frequent. She complained of pain and dizziness in the head, her face was florid, and her skin hot and dry.

" Upon inquiry it appeared, that the patient had been troubled with cough for several days; and she mentioned of her own accord, that she had observed an unusual palpitation at her heart for some time. Six weeks had elapsed, according to her account, since the last appearance of the menstrual evacuation. She had been costive, and entirely lost her appetite. Sixteen ounces of blood were drawn from her arm, muriatic acid was directed, and a blistering plaster was the next day applied to her side.

" During the month of March, the cough was very distressing through the night, but in the day-time not very frequent. It was quick, almost spasmodic. She expectorated, largely, thick frothy mucus. The palpitation of the heart could now be observed through her clothes by the bystanders at a considerable distance. It appeared by regular paroxysms, almost invariably at eleven in the morning, and at five or six o'clock in the evening. In the night, when awaked by coughing or frightful dreams, which frequently happened, it was most violent, and attended with such difficulty of breathing as to force force her to start up and remain with her body erect, until the paroxysm abated. She laid with her head raised very high by pillows, but said she breathed much more easily, when sitting in a chair. Pain in the side continued, and was occasionally severe. She became subject to frequent profuse sweatings at night, and, in the morning and at noon, to chills, followed by flushes of heat. Her pulse became more irregular, particularly in the paroxysms of dyspnœa and palpitation. It was generally frequent, but varied very much in hardness and fullness, and sometimes intermitted. Venesection and blistering gave some relief as before.

"In the two succeeding months her symptoms were highly aggravated. The cough became more violent, and the attacks of palpitation more distressing, particularly in the night time. She laid in bed with her head so much elevated by pillows, as to be almost upright. When she sat in a chair, she often rested her head upon her arms, supported by her knees. The palpitation of the heart was felt extending over a large part of the side, but perceived most distinctly at the epigastric region. It was accompanied with a constant sense of pain and distress in the whole course of the sternum, which she sometimes described as if a weight were laid over her heart, checking its motion. The carotids could be observed at a great distance pulsating very strongly. Her pulse became highly irregular, sometimes intermitting as often as once in 10 or 15 strokes. In the left arm it was usually slower and rather more contracted than in the right. Sometimes it was the bis feriens of authors. Large doses of hemlock and opium gave but little relief to the attacks of distress in the night, which were so severe as often to induce her to prefer sleeping in a chair to lying in a bed. Occasionally, but not often, hectic chills appeared in the morning. The perspirations in the night no longer continued profuse. She expectorated thick whitish mucus, generally mixed with large quantities of a clear pellucid fluid. When difficult it was often relieved by squills. Her appetite was tolerably good, and her bowels were extremely costive, probably from the use of opium.

"The violence of the paroxysms varied very much. One day she would feel comparatively easy and happy, as her breathing would be free, and the palpitations slight. The next, her symptoms would appear with renewed violence, and induce a state of absolute despair. Two attacks were so severe as to require venesection. Vesication over the sternum was kept up as constantly as possible.

"May 15th, her feet and ancles began to be swelled, and soon became œdematous. This appearance, however, after the assiduous use of friction with flannel, subsided in about ten days. On the 28th, in the afternoon, she became delirious, wildly rolling her eyes, tossing her limbs, recognising no one. She answered questions put to her, though confusedly, and complained of violent pain in her head. In the evening venesection was employed to give her relief, and while the blood was flowing from her arm, she became perfectly sensible. Her head continued dizzy, and she long complained of pain shooting through her temples. Blistering at the back of the neck, and behind her her ears, finally removed these troublesome symptoms, and for a fortnight before her death she remained perfectly free from them.

" On the 29th of June, her pulse became extremely small and frequent. The cough, and fruitless attempts to raise mucus from her throat, together with short and laborious respiration, gave her exquisite distress. In the night the palpitation was unusually violent, and the next morning at ten she expired.*

" *Dissection.*—The body was examined on the day after death. The countenance was quite livid. When the heart was opened, we were surprised at finding no appearance of disease in it, excepting a very moderate ossification of the coronary arteries; such as is often found in patients who die without a symptom of affection of the heart. The aorta was small in proportion to the heart. The pericardium contained one or two ounces of serous fluid. The lungs were universally in a state of induration, much resembling that of a scirrous breast. They yielded but little to pressure, and did not collapse in any degree when cut, nor their vessels, as usual, pour out blood. The cells contained a quantity of frothy mucus. No pus could be found. The pleura of the ribs was closely adherent to the lungs in every part, so that the thoracic cavity was completely filled by a resisting solid body.

" Each of the cases, related above, contains something worthy of particular remark.

" In the case of aneurism of the aorta, we find many symptoms, such as accompany disease of the heart, and yet an absence of some of the most important and characteristic. Among the former, are the difficult respiration, cough with copious expectoration, difficulty of lying in a horizontal posture, starting from sleep, and paroxysms of suffering with intervals of ease; but we do not observe the violent palpitations, the irregular pulse, and the watery effusions, which commonly attend diseases of the heart. How can we explain the absence of these appearances? Probably, the symptoms of disease, in this case, ought to be attributed rather to disturbance in the respiratory apparatus, than in the organs of the circulation. The pressure of a great tumor on the lungs would necessarily impede the exercise of their healthy functions, while it excited them, and irritated their vessels to increased secretion of mucus. Hence we should have difficulty of breathing, cough, and copious expectoration. But the canal of the aorta remaining open, no interruption existed to the discharge of blood from the heart; therefore no palpitations, no irregularity in the pulse, no impediment to the transmission of blood from the capillaries, and, of course, no effusion from the exhalants. If this explanation be just, it will follow that this case is precisely the reverse of case third; for, in the former, a disease in the organs of the circulation produced disturbance in the respiratory function, while, in the latter, a disease of the organs of respiration deranged the function of circulation. It is scarcely necessary to remark, that the difficulty in swallowing was

---

* " The notes of this case were principally taken by my late ingenious pupil, Mr. Henry Carnes."

caused by pressure on the œsophagus; and the acute pain in the shoulder and arm, by pressure on the first dorsal nerve, going to the brachial plexus, or by pressure on the phrenic nerve.

"The second case exhibits the terrible effects of a *sudden* change in the organization of the heart. It would be a deviation from the main object of this paper to inquire whether the diseased orifice, in the mitral valve, was the effect of rupture from some unknown cause; or whether it was the consequence of inflammation and ulceration. The thickened and tuberculated appearance of the edge of the orifice affords grounds for the latter suspicion; yet no traces of inflammation or ulceration could be discovered in any other part of the organ. The observations of M. Corvisart give us reason to believe that a rupture in the valve might occur suddenly, without previous disease, from a cause which the patient would not very readily disclose.

"The case of diseased lungs appears unintelligible at first view; for we observe in it the symptoms of diseased heart, without a correspondent change in the structure of that organ. It must be confessed, that, on the examination of the body of this patient, we were not a little disappointed and embarrassed; and our difficulties were not removed till lately, on meeting with a certain memoir of M. Portal, which has led us to a new view of this case, and to consider it as confirming, rather than subverting, the doctrine of pathognomonic symptoms of diseases of the heart. This memoir treats of the action of the lungs on the aorta during respiration; and is accompanied with the remarks of M. Bordeu, by which he endeavors to shew 'that the connection of the left bronchia with the aorta, may produce modifications in the pulse, that may be called pectoral modifications, or pectoral pulse.' It seemed probable, and even nearly certain, that, if the pressure of the bronchia on the aorta influenced the circulation of the blood, in a healthy state of organs, that this influence must be greatly increased in some diseases of the lungs.

"Before we inquire how such an influence could operate, we are naturally led to some investigation into the causes of the phenomena attendant on diseases of the heart. These seem principally to depend on disturbance in the organs of respiration and circulation; but the symptoms of disease in the respiratory organs evidently arise from disorder in the circulation of the blood, at least in most cases, as may be shewn presently. Our researches are, therefore, narrowed to an inquiry into the cause of disorder in the organs of the circulation. This cause seems to be a mechanical obstruction to the circulation of the blood as it passes through the heart or great artery; for, whether the disease be an induration of the auriculo-ventricular or aortal valves, or an aneurismal enlargement of the heart, there must generally be an obstruction to the passage of blood out of the heart, arising from disproportion between the quantity of blood to be transmitted, and the size of the passage to receive it. If the heart cannot discharge the whole, or at least the greater part, of its blood, that portion which remains must prolong the stimulus on the organ, or, rather, repeat it too suddenly. The heart, thus imperfectly stimulated, will contract imperfectly, with a tremulous motion, constituting palpitation. This tremulous motion, propagated along the blood in the arterial system, produces irregularity in the pulse. M. Corvisart informs us that the left side of the heart is more frequently diseased than the right, especially with ossification. If the blood be obstructed in its passage through the left side of the heart, it must be so in the pulmonary veins, and, of course, in the whole vascular system of the lungs. There accumulated, it compresses the air-cells, prevents the free admission of air, and excites difficult respiration, cough, and their concomitant symptoms. The copious discharge of mucus from the lungs, and, in the latter stages of the disorder, discharges of blood, proceed from the exhalant vessels of the lungs, which receive an unusual quantity of fluids from the capillary vessels, because the latter cannot freely empty themselves into the veins. Continuing to pursue the circulating system backward, we observe accumulation of blood in the jugular veins, and in the veins of the face and head, causing dizziness, intense head-ach, and purple color of the lips and face; we sometimes also observe such accumulation in the liver. The whole venous system seems overcharged with blood, which is, probably, the cause of the permanent dark color of the skin, observed in some violent cases. As the blood is collected in the venous system, it cannot readily be emptied by the general capillary system into the origins of the veins, it will therefore be thrown upon the exhalant vessels in every part of the body, and thence its thinner or serous portion will be poured into the cellular membrane, into the cavities of the abdomen, thorax, and pericardium. These explanations seem to flow very naturally from a little observation of the phenomena and morbid changes in organic diseases of the heart. They are, however, offered with diffidence, as results which have occasionally suggested themselves, and not as the consequences of any very profound research. They may also have been presented, at least in part, by those able hands into which the investigation of these diseases has fallen.

"If it should be admitted that the symptoms of diseases of the heart arise from a mechanical obstruction to the circulation of the blood through that organ, there will be no difficulty in explaining the appearances in our case of diseased lungs. A mechanical cause on the outside of the heart, or aorta, may certainly obstruct the passage of blood as much as a cause existing within. The lungs, transformed into a hard tumor filling nearly the whole thoracic cavity, might, we suppose, make such pressure on the aorta near the spine, or, perhaps, on some part of the heart itself, as to interrupt the passage of the blood. From that interruption would follow the symptoms of organic disease in the train we have pointed out.

"After examining the best writers on morbid anatomy, I have not been able to discover a case of similar disease of the lungs, whose symptoms corresponded with those of this case. That which approaches most nearly to it, is to be found in Lieutaud, who quotes it from De Haen, and is headed 'The heart falsely accused.' * 'On examining

---

* *Cor falsò incusatum.* Obs. 611. Lib. 2. Lustrato cadavere cujusdam examining the body of a certain little young woman who for many years encountered a violent palpitation of the heart, panting, anxiety about the præcordia, and frequent cough, in spite of various remedies, the vital organs were found to be perfectly healthy, if you except a genuine but very slight adhesion of the lungs to the pleura. Moreover, three worms were discovered in the intestine ilium.' "

Dr. Channing has communicated an interesting paper on the diseases resembling syphilis, which, however, we shall pass by, not having the first part of it in the number before us. The same cause also precludes us from entering upon the valuable communication of Dr. Warren, on apoplexy, in which, from the history of the attack, and the appearances on dissection, that able physician infers, that there are good reasons to be found in anatomy, physiology, and pathology, for believing that the stomach is capable of "materially affecting the head." To this inference we fully assent; and, when the stomach, in examinations of apoplectic subjects, shall be attended to with that minuteness of observation which is now principally directed to the brain, we doubt not that numerous instances will confirm our judgment, at present founded on very limited experience.

In a short account of the operations of lithotomy and aneurism, Mr. Astley Cooper is stated to have, of late years, made great use of the knife in lithotomy. The mode of operating is neatly described. The following paragraph will be new to most of our readers: "In France, Dubois, perhaps the most dexterous surgeon living, uses and recommends a double-edged scalpel (in lithotomy). He performs the operation with a rapidity which astonishes every one."

Dr. Martin, of Bangor, in the district of Maine, has related two cases of necrosis, which merit great attention. With them we shall close our account of the present number; and promise ourselves much satisfaction in being shortly able to notice the third number of this valuable work.

"Case I.—In March, 1810, I. W. aged 24, with blue eyes, and light hair and complexion, applied to me in a case of necrosis of the superior part of the humerus. He gave me the following account:

"About twelve years ago, he had a severe and tedious fever; and, soon after his recovery, had an acute pain in the arm, which seemed to lie deep in the bone. He in vain applied poultices, &c. &c. the

---

jusdam fœminæ juvenculæ, quæ multis abhinc annis, *vehementi cordis palpitatione, anhelitu, anxietate* præcordiorum, tussique frequenti, frustrà variis adhibitus præsidiis, conflictabatur; vitalia viscera sanitatem illibatam adeò referebant, ut nihil illibatius, si excipias genuinam, sed lævissimam, pulmonis adhæsionem ad plevram. Deprehendebantur insuper tres lumbrici in intestine ileo. *Clar. Haen.*

pain pain continuing till matter found a vent. His medical attendants treated the case variously: one sweated him; another starved and salivated him; while others used various injections; until one, more frank than the rest, told him that his disease was not within the reach of our profession, and that nature would either kill or cure him in seven years.

"I described to him his disease, and the method of cure. He left me doubting; and I did not hear from him till July 4, when he sent for me to operate.

"I made a single semi-circular incision (beginning about an inch from the head of the bone), dissecting the integuments and flesh back, so as to admit the trephine on the surrounding new bone. I found no necessity of wholly removing any of the integuments in the operation. The incision was about four inches in length, and ranged with a set of healed and fistulous ulcers, the whole arm seeming to have a tendency to ulceration. I extracted five inches of sequestra (or remnant of old bone), which was loose and moveable every way.

"There was no opening found through the surrounding new bone, larger than a crow-quill; but, by taking out a piece of the new bone, of the size of a half-crown piece (by means of Hey's saw, and a chisel), I was enabled to saw the sequestra into two parts, and to extract it. The arm was increased in size; the integuments were so universally diseased, as to present either the appearance of a fistulous ulcer, or of a cicatrix, and, in many places, their substance was connected in one common mass with the periosteum; the periosteum was much thickened, and adhered strongly to the new bone.

"The new bone was firm; and the patient said the arm had always been enough so for the ordinary uses of life, but had felt weaker than formerly about the commencement of the disease. When I cut through the new bone, it was about the sixth of an inch in thickness. The inner side, (that is, the inner parietes of the incasement,) had a coating which was, in general, smooth, but in some places granulated; and which, in some parts, was thick, but at others only formed a covering.

"The sequestra was absorbed in some places through its whole substance. Where it remained, it was hard and dry; in some parts, being left in the form of a honeycomb; and in others, as smooth as if the periosteum had just been cleansed from it. The absorption and non-absorption of the different portions of the sequestra, seemed to correspond to the quantity of granulations on the inner parietes of the surrounding new bone. The absorption from each end of the sequestra was so great, that I could only judge of the extent of the original sequestra by the enlargement of the integuments of the arm, which extended to near six inches and a half in length.

"The progress of the cure was uniform, being free from pain and foul discharge, and giving no more trouble than the dressing of a simple wound. It was near three months before the cure was perfect; since which the young man has been hearty, and has the perfect use of his arm.

"Case II.—The patient, I. Bailey, was 13 years of age. His father, having been present at the preceding operation, desired my opinion opinion concerning him. In consequence, I visited him, and found him just able to crawl about, having necrosis along the greater part of the tibia, from a blow on the shin about a year before.

"He had occasional pain; a small and quick pulse; no appetite; and night sweats. His whole leg seemed a mass of corruption, being full of ulcerous sinuses, leading to the affected bone. The discharge was profuse and foul, and some of the ulcers completely extended to the lower end of the tibia. The leg was very much enlarged, and a depending position produced pain.

"I deferred my decision on the case that day, hesitating between amputation and the extraction of the sequestra. On examining my books, I found little to confirm me in my hope of saving the limb. Reasoning, however, on the data which nature has given us in the cases where she has thrown off the whole bone, and finding that the patient's constitution had not only hitherto been his sole support, but that he had still, apparently, strength enough to go through an operation, while, on the other hand, he had a strong aversion to an amputation, I resolved to attempt the extraction.

"I made an incision on the inside of the leg, extending it each way, as appeared necessary for extracting the diseased bone.

"I found the periosteum much thickened and spongy; and in some parts almost incorporated with the integuments, (probably from previous inflammation.) I dissected it back, and found the new bone rough; and, in many places, perforated by vessels leading to the inner parietes of the incasement. The perforations were of a size capable of admitting a bristle freely. I then employed the trephine to obtain a passage through the new bone, which I explored in this way till I had removed the whole of the diseased bone within.

"The dead bone, at its upper part, was not entirely separate from the living. At the sides of the tibia, there was a considerable deposition of new bone, and some absorption from the old; but more along its middle line. The sequestra was wedged in very tight, being as yet, on the whole, not much absorbed. At the lower extremity, the old, and part of the new, bone, were so soft and spongy, that I cut into them with ease with my scalpel.

"I dissected the soft bony matter from the cartilage of the lower end of the tibia so extensively, as to leave but very little bone remaining for the support of the leg; the new deposition being destroyed by matter, before it had acquired solidity.

"While dissecting here, a sudden start of the patient made me cut into the cavity of the joint; and I found its internal appearance not only indicating no injury, but bearing the signs of perfect health.

"From the whole aspect, however, of the leg, I feared lest I might not save it. I was even apprehensive for the patient's life, as he was much exhausted, the operation having lasted a full hour. After dressing the wound as a simple one, and in the mildest manner, I gave the patient an opiate, and put him to bed. In the course of two hours, he complained of chills and head-ach, and vomiting began, which continued during four hours more. The next evening he was more composed, though some of the symptoms were still, in some degree, alarming. The day following, however, he was quite tranquil.

quil. He was confined to the bed but a few days, though to the house between fifteen and twenty. He soon regained his appetite, and his general health mended fast. He used crutches for nine months, but rather from choice than necessity, for he could bear his weight on the foot in three months after the operation, without the least uneasiness. When I took away his favorite crutches, he soon found a free use of his leg.

"He has now a limb that is perfectly sound, and as strong as the other; and the great vacancy left in the new bone is constantly growing less. Although his leg looks like the leg of a man placed upon the foot of a boy, and his joint is not quite so supple as that on the other side, his leg is a good one compared with one of wood.

"Nothing short of such perfect success, could have relieved my anxiety. "J. R. M."

